# Management of post-transplant lymphoproliferative disorders

**DOI:** 10.1097/HS9.0000000000000226

**Published:** 2019-06-30

**Authors:** Daan Dierickx, Vibeke Vergote

**Affiliations:** Department of Hematology, University Hospitals Leuven, Leuven, Belgium

**Take home messages**

- Reduction of immune suppression followed by rituximab has become the standard of care in the majority of post-transplant lymphoproliferative disorders.
- Currently pre-emptive therapy is only justified in allogeneic hematopoietic stem cell recipients.
- Adoptive immunotherapy is a very promising therapeutic modality, both in pre-emptive setting and as treatment of established Epstein–Barr virus-positive post-transplant lymphoproliferative disorder. However, use is still restricted because of labor-intensive procedure, reimbursement issues, and availability problems.

## Introduction

Post-transplant lymphoproliferative disorder (PTLD) constitutes a heterogeneous group of lymphoproliferative disorders increasing in medication-induced immunocompromised transplant recipients, including both solid organ transplantation (SOT) and allogeneic hematopoietic transplantation (HSCT).^1^ Although not required for diagnosis of PTLD, Epstein–Barr virus (EBV) plays a major role in the pathogenesis of a substantial proportion of cases. However, more recent reports show up to 50% of SOT-related cases are not associated with EBV.^2▪▪^ Established risk factors for PTLD development include EBV serological status at time of transplantation, type of transplant, and the type and intensity of immunosuppressive medication.^1^ In addition, there is growing interest in other potential contributing factors such as the role of the human leukocyte antigen system and of non-EBV viruses. Similar to other lymphomas, the gold standard for diagnosis is excision biopsy with histopathologic examination and categorization according to the World Health Organization 2017 classification.^3^ Despite their heterogeneity, about 85% of the PTLD cases are classified as CD20-positive diffuse large B-cell lymphomas (DLBCL). More in depth genetic-molecular research has increased our knowledge on pathogenesis of both EBV^+^ and EBV^−^ PTLD.^1^^,^^2▪▪^ In this article, we will focus on current treatment of both SOT- and HSCT-related PTLD, emphasizing recent advances in this field.

## Current state of the art

Several therapeutic options suppressing the malignant clone (T1), restoring the immune system (T2), and targeting EBV itself (T3) can be applied to PTLD patients (Table ^1^). Currently, there is insufficient evidence suggesting EBV^+^ and EBV^−^ PTLD should be treated differently, except of course in case of EBV-directed therapy.^4^

**Table 1:**
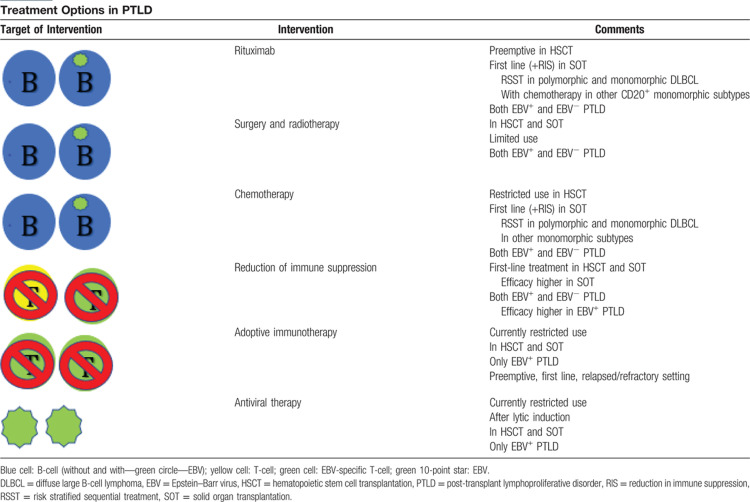
Treatment Options in PTLD

### Reduction of immune suppression (T2)

Reduction of immunosuppression (RIS) is considered the first but essential step in the treatment of SOT-related PTLD. In HSCT-related PTLD, immunosuppression is mainly the consequence of the conditioning regimen, making RIS at moment of PTLD diagnosis less important, although it should be applied when possible. Despite lack of hard evidence, most transplant physicians agree to reduce calcineurin inhibitor dose (at least 50%), discontinue antimetabolites, and continue steroids.^5^ However, this approach is not fully supported by results from clinical series and is in particular challenged by a recent retrospective analysis of the prospective PTLD-1 trial, suggesting corticosteroid use after PTLD diagnosis is associated with an increased relapse rate, whereas the use of antimetabolites is not.^6^ This discrepancy mainly reflects the difficult balance between primary focus on graft outcome versus lymphoma relapse risk. If possible, re-evaluation should be performed 2 to 4 weeks following RIS initiation. Response rates to RIS alone have a very wide variation ranging from 0% to >50% and are higher in EBV^+^ compared with EBV^−^ (often late onset) PTLD.^1^

### Rituximab (T1)

The monoclonal anti-CD20 antibody rituximab, combined with RIS, has become the standard of care for the majority of CD20^+^ cases, in particular polymorphic and monomorphic DLBCL subtypes, showing overall response rates (ORR) and complete responses (CR) in 44% to 79% and 20% to 55%, respectively.^1^ In the international prospective phase II PTLD-1 trial (restricted to SOT-PTLD), a risk stratified sequential treatment (RSST) approach was introduced. Patients with CR after rituximab induction received 4 additional courses every 21 days. In this subpopulation, estimated progression-free survival and overall survival at 3 years were 89% and 91%, respectively.^7▪▪^ Based on these data, RSST is currently considered the standard of care in newly diagnosed SOT-related polymorphic and monomorphic DLBCL subtypes. In HSCT, responses up to 65% have been described with rituximab, although preemptive strategy (RIS and rituximab based on EBV viral load in peripheral blood) is associated with improved outcome.^8▪▪^

### Chemotherapy with or without rituximab (T1)

Patients not or inadequately responding to RIS and rituximab need additional treatment, in most cases polychemotherapy. In the first part of the PTLD-1 trial (sequential treatment), all patients received 4 cycles of CHOP. In the RSST part, 4 cycles of rituximab-CHOP were administered in patients without CR after rituximab induction. Taken together, ORR was 88% (70% CR) with treatment-related mortality of 8%.^6^ In HSCT-PTLD data on chemotherapy are limited, showing a high treatment-related morbidity and mortality. Hence, chemotherapy is not recommended in first line.^8▪▪^^,^^9^

It is important to emphasize that most other histological subtypes require upfront specific chemotherapeutic treatment (combined with RIS and rituximab if CD20-positive), with markedly improved outcome for patients with rare PTLD subtypes.^3^,^10^,^11▪▪^

### Surgery and radiotherapy (T1)

The application of local therapy by means of surgery or radiotherapy in treatment of PTLD is limited to some rare specific situations, including treatment of local disease (with RIS), symptomatic control, palliative care or as part of a combined (immuno-)chemoradiotherapeutic approach.^1^

### Adoptive immunotherapy (T2)

Based on the high efficacy of unselected donor lymphocyte infusions in HSCT-PTLD,^12^ attempts were made to isolate EBV-specific cytotoxic lymphocytes (CTLs) aiming to induce a strong EBV-specific cellular immune response without risk of graft-versus-host disease. Both autologous (recipient-derived PTLD) and allogeneic (isolated from the donor itself or from a bank of partial human leucocyte antigen-matched voluntary donors) CTLs, targeting specific immunogenic EBV antigens, can be used.^13^ In a large multicentric study, 114 HSCT patients were treated with EBV-CTLs, either prophylactically or therapeutically.^14^ More recently, a Chinese prospective study in HSCT recipients demonstrated an increase in CR rates in patients treated with sequential administration of rituximab and EBV-CTLs.^15▪▪^ Despite very high response rates and excellent tolerance, wide applicability has been limited so far due to several reasons.

### Antiviral therapy (T3)

As most EBV-related lymphoproliferative disorders do not express viral protein kinase, monotherapy with nucleoside analogs failed to induce responses in EBV^+^ PTLD. However, pharmacological induction of viral thymidine kinase by administration of the histone deacetylase inhibitor arginine butyrate, followed by antiviral therapy, has shown promising results in a small pilot trial with an acceptable toxicity profile.^16^ More recently, there seems to be renewed interest in induction of lytic activation, which can be accomplished by several immunomodulatory drugs (immunomodulatory like lenalidomide) or proteasome inhibitors (in particular bortezomib).^17^,^18^

## Future perspectives

Despite increasing knowledge in pathogenesis of PTLD, incorporation of new treatment modalities—in particular targeted and personalized medicine—has lagged behind in PTLD compared to more common lymphoma subtypes. Important contributing factors for these factors include (a) exclusion of PTLD patients from clinical trials with new therapeutic approaches, (b) infectious vulnerability of transplant patients, and (c) possible risk of (fatal) graft rejection or graft-versus-host disease. However, recent pathological and molecular findings have identified promising pathways and surface receptors to be targeted, including BTK inhibition, PI3K and mTOR inhibition, anti-CD30 monoclonal antibodies, proteasome inhibition, and checkpoint inhibition (PD1-PD1-L pathway), which need to be tested in large international clinical trials.^1^,^2▪▪^,^17^,^18^,^19^,^20^ In addition, predictive and prognostic biomarkers need to be identified to optimize both prevention and treatment of PTLD.

## Acknowledgments

The authors would like to recognize and thank the other members of the “Leuven PTLD consortium”: Prof. Dr. Gregor Verhoef (Department of Hematology), Prof. Dr. Thomas Tousseyn (Department of Pathology), and Prof. Dr. Olivier Gheysens (Department of Nuclear Medicine) with whom we collaborate for all PTLD focused research.

DD holds Mandates for Clinical and Translational Research from the University Hospitals Leuven and from “Kom op tegen Kanker.” DD is a co-founder of “Stefanie's Rozen Fonds,” “Fonds Tom Debackere voor lymfoomonderzoek,” and “Fonds Jos en Mieke Vandevordt-Gaul voor de pathogenese van zeldzame lymfomen,” promoting research on rare aggressive lymphomas. VV is a PhD student, financially supported by a Mandate for Clinical Research from the University Hospitals Leuven.
